# Supplementary material for: Identification of photoperiod-induced specific miRNAs in the adrenal glands of Sunite sheep (Ovis aries)
Source: Front Vet Sci. 2022 Jul 22;9:888207. doi: 10.3389/fvets.2022.888207 (PMC9354845; doi:10.3389/fvets.2022.888207)
Supplement: Supplementary file 1 [file Data_Sheet_1.ZIP › Supplementary file/miRDeep2 file.zip!/miRDeep2 file/Know_Structure/SP42A3/oar-mir-370.pdf]

miRBase precursor : oar-mir-370
Total read count : 5702
oar-miR-370-5p read count : 24
oar-miR-370-3p read count : 5668
remaining reads : 10

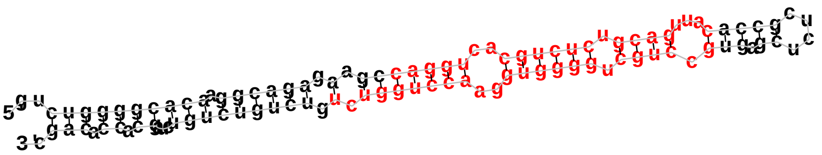

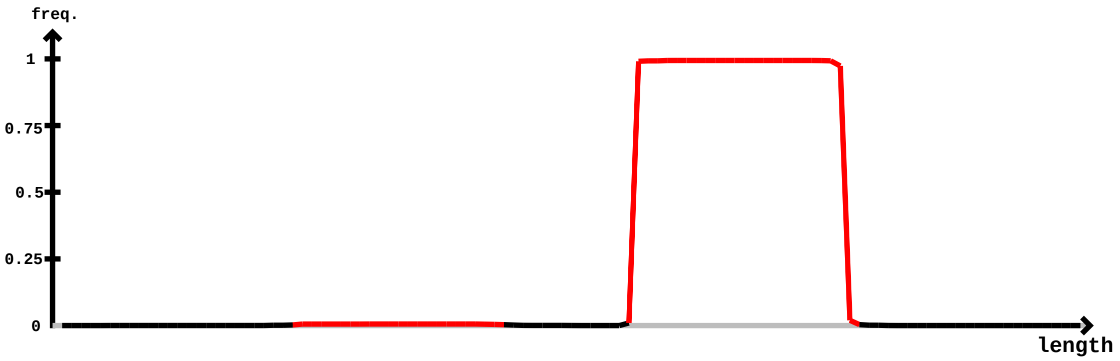


```
                                                                                  oar-miR-370-3p
                            oar-miR-370-5p
5'- gucuggggcacaaggcagagaagccaggucacgucucugcaguuacaccgcucucgagugccugcuggggguggaaccuggucugucugucugucuagcaccacagc   -3'    exp
    ..((((((((((.((((((.(.(((((((..((((((.((((...(((((....)).))).)))).))))))..))))))).).)))))))))...)).)).))).      reads      mm         sample
    ....ggggcacaaggcagagaagc...................................................................................      1          0          seq
    .....................agccaggucacgucucugcagu................................................................      3          0          seq
    .....................agccaggucacgucucugcaguu...............................................................      2          0          seq
    .....................agccaggucacgucucugcaguua..............................................................      1          0          seq
    .......................ccaggucacgucucugcagu................................................................      1          0          seq
    .......................ccaggucacgucucugcaguu...............................................................      1          0          seq
    .......................ccaggucacgucucugcaguua..............................................................      3          0          seq
    .......................ccaggucacgucucugcaguuaca............................................................      1          0          seq
    ........................caggucacgucucugcaguu...............................................................      1          0          seq
    ........................caggucacgucucugcaguua..............................................................      2          0          seq
    ........................caggucacgucucugcaguuac.............................................................      8          0          seq
    ........................caggucacgucucugcaguuaca............................................................      6          0          seq
    ........................caggucacgucucugcaguuacac...........................................................      1          0          seq
    ........................caggucacgucucugcaguuacaccgcu.......................................................      2          0          seq
    ...............................cgucucugcaguuacaccgcuc......................................................      1          0          seq
    ..........................................................ugccugcuggggguggaaccugg..........................      4          0          seq
    ..........................................................ugccugcuggggguggaaccuggu.........................      50         0          seq
    ..........................................................ugccugcuggggguggaaccugguc........................      2          0          seq
    ...........................................................gccugcuggggguggaaccu............................      2          0          seq
    ...........................................................gccugcuggggguggaaccug...........................      5          0          seq
    ...........................................................gccugcuggggguggaaccugg..........................      106        0          seq
    ...........................................................gccugcuggggguggaaccuggu.........................      5384       0          seq
    ...........................................................gccugcuggggguggaaccugguc........................      89         0          seq
    ...........................................................gccugcuggggguggaaccuggucu.......................      8          0          seq
    ............................................................ccugcuggggguggaaccuggu.........................      4          0          seq
    ............................................................ccugcuggggguggaaccugguc........................      1          0          seq
    ............................................................ccugcuggggguggaaccuggucu.......................      4          0          seq
    .............................................................cugcuggggguggaaccugguc........................      1          0          seq
    ..............................................................ugcuggggguggaaccuggucug......................      1          0          seq
    ..............................................................ugcuggggguggaaccuggucugu.....................      5          0          seq
    ..............................................................ugcuggggguggaaccuggucuguc....................      1          0          seq
    ...............................................................gcuggggguggaaccuggucugu.....................      1          0          seq
```
